# The Spread of Syphilis by Cigars

**Published:** 1892-05

**Authors:** W. S. Gottheil


### THE SPREAD OF SYPHILIS BY CIGARS.

The writer reports the cases of two girls with distinct syphilitic lesions, who were employed in cigar factories, as "finishers." They used to bite off the ends of the cigar wrappers, using the saliva to shape the tips. Gottheil calls attention to the manifest dangers in this process. He is not aware that specific disease has actually been communicated in this way. It is possible that the tobacco leaf and tobacco juice in the mouth may render the contagious element innocuous. But it is also possible that the long period of incubation of syphilis has rendered it impossible to trace a source of contagion so unnoticeable. It remains a fact that upon every single cigar tip of the thousands finished by these two operatives, there was probably deposited a portion of the virus of the disease. Moreover, the practice of using the teeth and saliva in the manufacture of an article which is destined to be taken into the mouth is not without serious objections entirely apart from considerations of disease.

It will probably be impossible in the future, as it has been in the past, to stop this unclean and dangerous method of cigarmaking by pressure put on the operatives themselves. The saving time and trouble is so great as to outweigh every other consideration.—*W. S. Gottheil in N. Y. Med. Jour.*, Mar. 19.—*Epitome of Medicine.*

